# Oral Health-Related Quality of Life of Greek Adults: A Cross-Sectional Study

**DOI:** 10.1155/2011/360292

**Published:** 2011-09-05

**Authors:** William Papaioannou, Constantine J. Oulis, Demetra Latsou, John Yfantopoulos

**Affiliations:** ^1^Department of Preventive and Community Dentistry, School of Dentistry, University of Athens, 2 Thivon St., 115 27 Athens, Greece; ^2^Department of Paediatric Dentistry, School of Dentistry, University of Athens, Athens, Greece; ^3^School of Law, Economic and Political Sciences, University of Athens, Athens, Greece

## Abstract

*Purpose*. The aim of the present study was to investigate the impact of oral health status on the quality of life of adults in different regions of Greece, using the Oral Health Impact Profile-short form (OHIP-14). *Methods*. A random sample consisting of a total of 504 Greek adults between the ages of 35–44 years (mean 39.1 ± 3.5) was selected from different urban and rural areas, and face-to-face interviews were conducted using the validated Greek language OHIP-14. Associations of the total OHIP-14 score and its 7 sub-scales along with the self-perceived quality of life were evaluated with Spearman's correlations. *Results*. The subjects had an overall weighted OHIP-14 score of 1.1 (sd 1.9). No significant differences were found for either rural or non-metropolitan areas when compared to urban or metropolitan regions. High scores of above 2 were determined for functional limitation, physical pain, handicap, and the psychological discomfort scales. The education level of the subjects had a significant positive impact on the quality of life of the subjects. *Conclusions*. Dental and oral health conditions are factors that do impact on the quality of life of individuals.

## 1. Introduction

Health-related quality of life is an emerging subject of importance during recent years. This is based on the realization that the effects of a disease or condition cannot be fully determined by using solely clinical measures, since these do not take into consideration the subjective experiences that individuals have concerning their health [1]. Quality of life measures are of importance in order to look further than just the presence of health or disease and into the way that the individual perceives his state of health and how this impacts on his daily performance. Moreover, this is of importance when considering health care and how it is provided to different groups and if it is according to the specific needs they may have. 

The quality of life a person may enjoy, in general, as well as the factors that contribute to it varies according to differences in age, gender as well as cultural differences [2]. The impact of an individual's health on his/her daily activities can be significant. Actions and interactions can be greatly affected which in turn may further reduce the individual's functionality and psychological well being. Thus, health-related quality of life (HRQoL) is considered a multidimensional concept, which refers not only to patients' physical well being but also to their psychological and social well being. It is widely recognized for the assessment of healthcare outcomes. One of the major factors that can significantly impact on the overall HRQoL is the oral health of the individual [3, 4]. 

Oral health is an integral part of the overall health of all individuals. It impacts on the daily function and well being of an individual, leading even to the possibility of incapacitating him/her either physically or psychologically. Indeed, it is becoming accepted that problems in oral health can create significant complications and cost not only for the specific individual but also for society as a whole [5]. This applies to all societies in general. Thus, it is for the overall good of both the individual and society that quality of life is taken into account.

The last two decades have seen the development of a large spectrum of HRQoL measures in the field of dentistry, in an effort to capture the impact of oral disorders both on patients' physical and psychological as well as social well being and their ability to perform daily activities [6, 7]. From these, the oral health-related quality of life (OHRQoL) measurements have been developed.

The oral health status can in turn be affected by many personal, social, and local factors. Differences in the oral health status can indeed be seen when comparing differing regions within a country or between countries and geographical locations. Indeed, in a recent epidemiological study in Greece, significant differences were found between different regions and between urban and rural (agricultural) areas for the oral status of the inhabitants of these regions [8]. 

One of the most widely known OHRQoL instruments, the oral health impact profile in its short form (OHIP-14), was developed by Slade and Spencer (1994) [9] for the measurement of disability and discomfort due to oral conditions. It comprises 14 items which were derived being from the original 49-items version [10]. These items are subsequently transformed into seven subscales based on a conceptual oral health framework suggested by Locker (1988) [11] and derived from the World Health Organization (1980) [12]. The subscales based on the impact of the teeth mouth or dentures are (1) functional limitation (difficulty pronouncing words and/or worsened sense of taste), (2) physical pain (aching in the mouth and/or uncomfortable to eat), (3) psychological discomfort (self-conscious feeling and/or tense feeling), (4) physical disability (unsatisfactory diet and/or need to interrupt meal), (5) psychological disability (difficulty to relax and/or being embarrassed), (6) social disability (irritability toward other and/or difficulty performing ones usual job), and (7) handicap (feeling that life was less satisfying and/or being totally unable to function). The OHIP-14 is less time consuming and more practical, thus, preferable while a wide range of studies have shown it to have comparable reliability and validity [10, 13] with the long version. 

The aim of the present study was to investigate the impact of oral health status on the quality of life of a cross-section of adults aged 35 to 44 years old belonging to different population groups in different regions of Greece. This is a part of a larger study investigating the quality of life in different age groups.

## 2. Material and Methods

The study was conducted in different regions of Greece. A random sample consisting of a total of approximately 500 Greek adults between the ages of 35–44 years (mean 39.1 ± 3.5) were selected from different urban and rural areas. The discrimination of the individuals according to the different regions was based on international standards and the last census of 2001 of the *Hellenic Statistical Authority (El.Stat.).* Moreover, in order to be able to make comparisons with the findings of a previous epidemiological study [8], subjects were selected from the following counties: Attica and Thessaloniki, which are the two having the major metropolitan centres, Athens and Thessaloniki (where the majority of the Greek population is situated), and 3 counties with non-metropolitan cities, specifically Achaia (Patras), Ioannina, and Kastoria. Consequently, from the above-mentioned regions of Greece, a distinction was made between inhabitants of urban and rural areas. Stratified cluster sampling was used in order to obtain representation of diverse population groups which may have different quality of life. The subjects came from a random choice of meeting points and places of work according to the peculiarities of the different groups and sample regions.

 The study was approved by the Committee for Ethics and Research of the Athens Dental School. All participating subjects provided informed consent after being acquainted with the purpose. A self-administrated questionnaire was designed, and face-to-face interviews were conducted by one dentist trained in OHRQoL terms between October 2007 and September 2009. Participants were asked to evaluate on a 5-point Likert scale (0 = never, 1 = hardly ever, 2 = occasionally, 3 = fairly often, and 4 = very often) how frequently during the last year had experienced any of the problems assessed by the validated 14-item OHIP, while data regarding their sociodemographic profile (e.g., information concerning age, sex, education level, and occupation) were also recorded. Specifically for the occupation, the distinctions were self-standing business, private employee, public employee, retired, unemployed, and housewife.

As previously mentioned, the short form of the oral health impact profile or (OHIP-14) is one of the most widely known OHRQoL instruments. Apart from the general score, the results can be broken down into the 7 subscales which represent the different facets of or impacts of oral health. These subscales are psychological disability, social disability, handicap, physical disability, physical pain, functional limitation, and psychological discomfort. The OHIP-14 has been validated for the Greek language for both adolescents and adults [14].

Additionally, the questionnaire included items for the assessment of different types of construct validity given the absence of a universally accepted “gold standard.” More specifically, data regarding self-perceived general and oral health status were taken into consideration, as well as participants' satisfaction with their oral health status.

The associations between the OHIP-14 score and its 7 subscales with the self perceived quality of life were evaluated with Spearman correlations. Comparisons that were made were between metropolitan *versus *non-metropolitan, rural *versus *urban, and the self-perceived health status (both oral and general).

## 3. Results

A total number of 504 adults were finally interviewed. From the 2 major metropolitan areas, the numbers were for Athens a total 104 subjects of which 2 were considered to live in rural conditions while for Thessaloniki and a total of 100 subjects 18 were considered to live in rural conditions on the outskirts of the city. For Patras, total subjects 100 with 24 rural, Ioannina 100 total with 53 rural and Kastoria from a total of 100 those from rural areas numbered 22.

Concerning the gender of the sample population, 49.8% were men while the women comprised the 50.2%. From the total sample, 53.8% reported to be smokers. Another factor that could affect the responses to the question is the educational level. The majority of 41.4% reported to have finished the middle level of education, 22% higher education, and 21.8% highest. Those reporting lower as well as postgraduate education were in the minority (4.4% and 10.4%, resp.). For the occupation of the individuals, almost equal proportions were self standing (29.6%), private employees (29.4%) and public employees (33.3%) while a very small proportion of this population (0.2%) reported to be retired. The remaining comprised of housewives (6.3%) and unemployed (1.1%).

**Figure 1:**
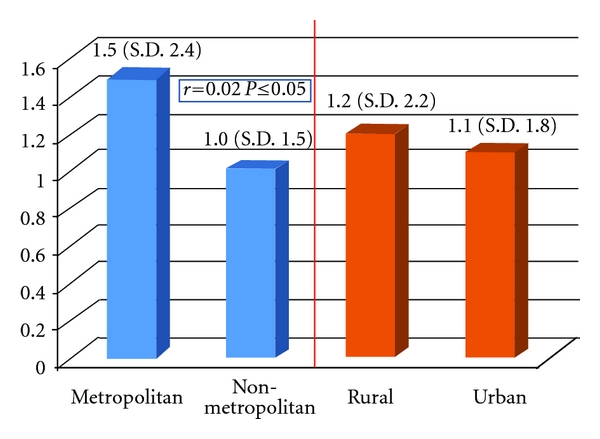
The mean OHIP-14 score (SD) according to region. Correlations and significances are shown.

**Figure 2:**
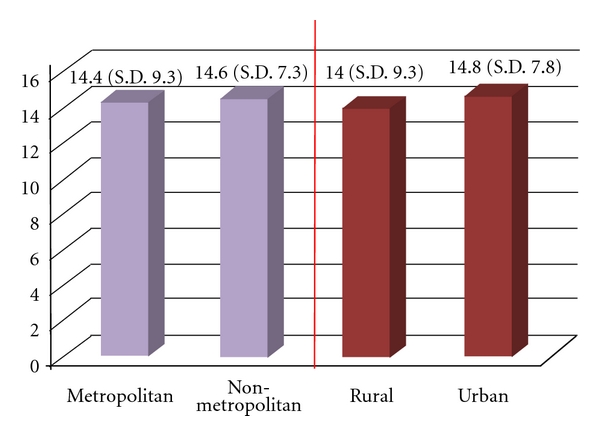
The mean additive OHIP-14 score (SD) according to region.

Internal reliability was tested and returned a very good internal consistency with a Cronbach alpha of 0.88. The subjects overall had a weighted OHIP-14 score of 1.1 (sd 1.9). The metropolitan subjects had a higher score when compared to the non-metropolitan (1.5 versus 1.0, resp.); however, this difference was almost non existent when the subjects were distinguished as rural or urban (Figure 1). A weighted score above one means that the overall quality of life was slightly affected by oral health. This diminished impact is also revealed if the results are shown with the additive method (Figure 2). The metropolitan subjects showed a slightly lower impact of their oral health on their quality of life (14.4 mean additive score versus 14.6 for the non-metropolitan subjects). Thus, this score was not found to have a significant correlation with either the metropolitan/non-metropolitan distinction or the rural/urban.

For the seven subscales of the OHIP-14 tool (Tables 1 and 2), only one was found to have a significant correlation for the inhabitants of the different areas (Table 4). Specifically, an important and significant correlations was discovered for functional limitation (*P* < 0.01), for the urban rural distinction. This subscale tended to have the highest scores (in the area of, and reaching even, 3) in comparison to the others. The trend discovered was for a higher impact in inhabitants of the more rural areas. All the other subscales did not differ between the different regions and cities.

Along with the functional limitation, the psychological discomfort subscales also reached a relatively high score (*≈*3) as was reported for the different cities without, however, important differences between cities or the types of regions. Physical pain and handicap also surpassed the level of score 2.

Concerning the OHIP-14 score and the gender differences, the scores were found to be at a similar level for the two genders, with the male 1.8 (SD = 1.9) and female 1.2 (SD = 2.0). Only 1 of the 7 subscales was found to be significantly different between genders. The handicap subscale, with the males at 2.3 (SD = 5.2) female 2.2 (±1.5) (*P* ≤ 0.05).

Correlations were found between the OHIP-14 scores and 2 of its subscales, with the education level (*P* ≤ 0.05). Thus, as the education level increased the impact on the total score, the social disability (scale 6) and handicap (scale 7) scales decreased. Alternatively, no correlations were found between the OHIP-14 scores and any of the 7 subscales with the occupation of the subjects.

**Figure 3:**
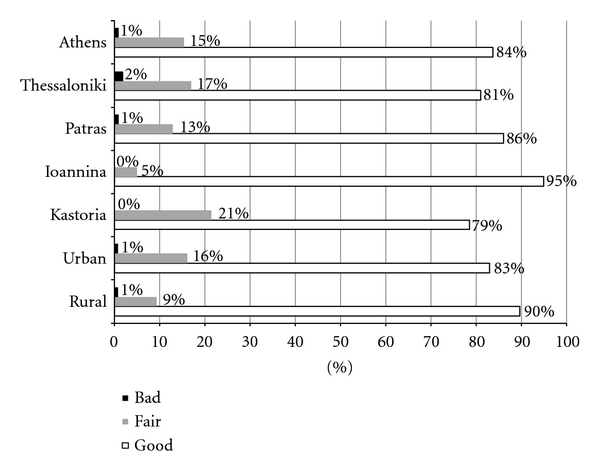
Proportion of responses to the question “How would you judge your general health?”.

**Figure 4:**
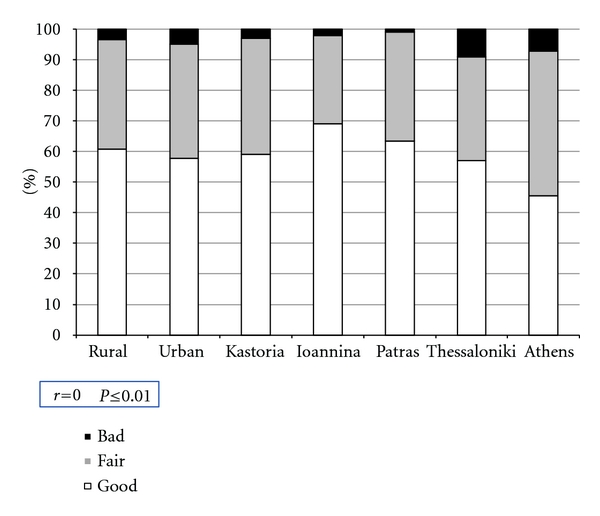
Proportion of responses to the question “How would you judge your oral health?”.

The majority of subjects in both rural and urban regions considered their general health to be good (90% and 83%, resp.), while only 1% as bad. This is mirrored in all the 5 areas (Figure 3) with the majority having a positive opinion. Concerning oral health (Figure 4), approximately 60% of the subjects in both rural and urban areas judged this as being good, while only 3.4% and 5% as bad, resp. When regions are examined, the adults from Athens had the lowest percentage for self-perceived oral health as being good, 45.2%, while Thessaloniki had the highest percentage (9%) reporting bad oral health. Significant correlations were found (at the *P* ≤ 0.01 level) for the OHIP-14 score with both self-reported oral and general health. The majority (77.6%) considered their general health to be good which was reflected in their mean ohip score of 0.9 (SD = 1.6). For oral health, the number considering it to be good was greatly lower (54.2%) than for general health. For oral health, a number of subjects (4.2%) even reported it as being bad, and these individuals had a mean OHIP-14 of 4.4 (SD = 3.7).

For the question “*are you satisfied with your oral health?*” a significant correlation (*P* ≤ 0.01) was determined for the score with oral health satisfaction. The majority of the subjects (65.1%) was satisfied and thus had a low impact on their quality of life (mean OHIP-14 score of 0.6, SD = 1.3). Those that were not satisfied with their oral health (34.1%) had a higher OHIP-14 score of 2.2 (SD = 2.5).

And finally for the question “*how does your oral health compare to others?*” a significant correlation (*P* ≤ 0.01) of the OHIP-14 score was determined with how each individual perceives his oral health against his peers. An almost equal proportion answered “better” or the “same” 43.5% and 42.5%, respectively, but 11.7% considered their oral health to be worse. The impact score increases quite significantly over the 3 answers (0.7, 1.0 and 3.6 for better, same, and worse, resp.). 

When the subjects are grouped according to their education level (Table 3), the subscale of quality of life that is most affected by problems in the oral condition is that of functional limitation. For every education level bad oral health significantly affects good function. It is important also to note that the higher the education level the lower the overall OHIP-14 score, going from a high of 1.9 for the lowest level to 0.8 for the postgraduated level. Moreover, the lowest level shows a mean score of over 2 for 5 out of the 7 subscales, surpassing the other levels.

## 4. Discussion

The present study was a population-based study using the short form of the OHIP with 14 standardized questions that has previously been translated into the Greek language and tested for validity [14]. The internal consistency found for the present study with a Cronbach *α* of 0.88, greatly exceeds the minimum recommended level for this instrument. The sample was chosen to be representative of Greek population in this specific age group of 35–44 years old. This is the first study to examine this section of the Greek population concerning oral health quality of life. 

Just as our study on the adolescent population (unpublished data) as well as other studies [15], the impact of oral health is limited for youngsters and young adults. Scores above 1 mean that there is an impact of oral health on the overall quality of life. The present study showed a weighted OHIP-14 score of 1.1 meaning a relatively low effect confirming the previous studies. This is relatively low score in comparison to other similar studies with adults [16, 17]. The subscales with the major impact, especially concerning psychosocial impact, are similar to those that John and co-workers (2004) [18] reported although the age range they examined was significantly greater (16–79 yrs.).

The question arises as to why the impact of oral health seems to be quite low and comparable to that of the adolescent group? A possible explanation could be that the severe periodontal disease and tooth loss which can increase problems in function and impact on an individual's quality of life are usually at an older age [19]. In an adult Swedish population, the highest OHIP score was associated with severe tooth loss [17], something that was probably very low in the present group. Indeed, the Eurobarometer on oral health [20] reports that the number of individuals in Greece stating that they still have all their natural teeth is relatively high and comparable to northern European countries. Unfortunately, the study was not paired with a clinical examination of the subjects. However, when the self-perceived level of health for the subjects of this study was considered as being bad or worse than their peers, the impact also increased significantly reaching even a score of 3.6. A low perception of oral health led to also a higher impact on the quality of life of the individual, emphasizing the validity of the OHIP-14 questionnaire. This perception can, however, be affected, apart from the actual oral condition, by the general outlook of a person which by general admission tends to be less pessimistic in younger individuals than older adults and thus would probably tend to overlook less important problems with their oral health. In this group, contrary to what was seen with the adolescents, the metropolitan subjects had a higher score when compared to the non-metropolitan (1.5 versus 1.0, resp.). This again can probably be attributed again to possible differences in oral health level between the two regions or possibly to a slightly less positive outlook in life in general for the city dwellers. 

Indeed, by comparing the present results with those from the Hellenic pathfinder survey [6] in Table 5, this difference in general life outlook for the dwellers of the capital is clear. The subjects from Athens, for the specific age group have been shown to have the lowest DMFT score, including the lowest number of missing teeth (MT), in comparison with the 4 other cities. However, if the subscales of functional limitation handicap and psychological discomfort are examined, these subjects from the capital region show the highest impacts. This is especially true for the feeling of handicap. Conversely, Ioannina which presents the highest DMFT and missing teeth showed in the present study the lowest impacts for the aforementioned subscales. It would seem that the people living in the mountainous region of Epirus in the north of Greece are not easily concerned by the presence of caries or even of missing teeth. This finding could also indicate a possible weak point in the OHIP-14, in that it may not adequately factor in the difference in expectations that may exist between different regions. This in turn may result in erroneous measurements of the oral health impact on the quality of life, either by under stressing or even overstressing it according to the population examined.

The fact that there was a distinct education level effect on the reported quality of life of the individual is in confirmation with the European wide study which reported similar findings. Namely, the higher the standard of living of an individual the fewer problems he/she encountered with their teeth [20], which as shown in this study is not necessarily linked to the type of employment. 

The oral health impacts on the quality of life of the individual's, though of a low impact, were not completely negligible even for such a general population study. It would be of great interest to further investigate the Oral health quality of life in individuals focusing on those with significant signs of disease, present or past. Indeed, the major limitation of the present study is the lack of data concerning the actual clinical situation (presence of caries and/or periodontal disease, missing teeth, etc.) of the subjects. It must be stressed again, that these data are necessary in order to be able to efficiently advocate for resources as well as to allocate public funds and resources in dentistry [21]. It is important to place dental and oral health in the proper context and to show the powers that this factor affects the ability to function which in turn has more far reaching economic ramifications.

## 5. Conclusions

1. Functional limitation, handicap, physical pain, and psychological discomfort were the primary dimensions affecting the QoL of the subjects.
2. Subjects from metropolitan regions had lower oral health related quality of life (OHRQoL) compared to those in non-metropolitan.
3. A reduction of the OHIP-14 was seen as the level of education increased.

## Figures and Tables

**Table 1:** Mean OHIP-14 for the subscales and total scores according to city.

City	Functional limitation	Physical pain	Psychological discomfort	Physical disability	Psychological disability	Social disability	Handicap	OHIP score	OHIP add
Athens	3.0 ± 1.6	2.4 ± 1.5	2.9 ± 1.6	2.0 ± 1.7	1.5 ± 1.9	1.1 ± 1.5	2.9 ± 7.9	1.5 ± 2.5	15.3 ± 8.9
Thes/niki	2.8 ± 1.9	2.1 ± 1.7	2.9 ± 2.1	1.7 ± 2.1	1.1 ± 1.6	0.7 ± 1.5	1.8 ± 1.6	1.4 ± 2.2	13.6 ± 9.9
Patras	3.2 ± 1.4	2.1 ± 1.4	2.8 ± 1.5	1.7 ± 1.5	1.6 ± 1.5	1.0 ± 1.2	2.1 ± 1.3	1.1 ± 1.7	15.0 ± 7.0
Ioannina	2.4 ± 1.4	2.0 ± 1.3	2.8 ± 1.2	1.8± 1.3	1.5 ± 1.6	1.0 ± 1.3	1.9 ± 1.1	0.8 ± 1.3	14.0 ± 6.7
Kastoria	3.0 ± 1.5	2.1 ± 1.4	2.9 ± 1.7	1.9 ± 1.6	1.7 ± 1.4	1.2 ± 1.3	2.2 ±1.7	0.9 ± 1.6	15.0 ± 8.3

**Table 2:** Mean OHIP-14 for the subscales and total scores according to region.

Region	Functional limitation	Physical pain	Psychological discomfort	Physical disability	Psychological disability	Social disability	Handicap	OHIP score	OHIP add
Urban	3.0 ± 1.5	2.2 ± 1.4	2.9 ±17	1.9 ± 1.7	1.5 ± 1.6	1.0 ± 1.3	2.3 ± 4.3	1.1 ± 1.8	14.8 ± 7.8
Rural	2.5 ± 1.8	2.0 ± 1.7	2.8 ±1.6	1.8 ± 1.7	1.5 ± 1.7	1.0 ± 1.6	2.0 ± 1.5	1.2 ± 2.1	14.0 ± 9.3

Metro	3.0 ± 1.8	2.3 ± 1.6	3.0 ± 1.9	1.9 ± 2.0	1.3 ±1.8	1.0 ±1.5	2.4 ± 6	1.5 ± 2.4	14.4 ± 9.3
Nonmetro	2.9 ± 1.4	2.1 ± 1.4	2.9 ± 1.5	1.9 ± 1.5	1.6 ±1.5	1.1 ±1.3	2.1 ± 1.4	1.0 ± 1.5	14.6 ± 7.3

**Table 3:** Mean OHIP-14 for the subscales and total scores according to educational level.

Educational level	Functional limitation	Physical pain	Psychological discomfort	Physical disability	Psychological disability	Social disability	Handicap	OHIP score	OHIP add
Elementary	3.0 ± 1.6	2.4 ± 1.5	2.9 ± 1.6	2.0 ± 1.7	1.5 ± 1.9	1.1 ±1.5	2.9 ± 7.9	1.9 ± 2	20.5 ± 9.8
Middle	2.8 ± 1.9	2.1 ± 1.7	2.9 ± 2.1	1.7 ± 2.1	1.1 ± 1.6	0.7 ±1.5	1.8± 1.6	1.0 ± 1.9	14.7 ± 8.0
High School	3.2 ± 1.4	2.1 ± 1.4	2.8 ± 1.5	1.7 ± 1.5	1.6 ± 1.5	1.0 ±1.2	2.1 ± 1.3	1.2 ± 1.9	14.9 ± 8.3
University	2.4 ± 1.4	2.0 ± 1.3	2.8 ± 1.2	1.8 ± 1.3	1.5 ± 1.6	1 ±1.3	1.9 ±1.1	1.1 ± 2.0	14.0 ± 8.3
Post-graduate	3.0 ± 1.5	2.1 ± 1.4	2.9 ± 1.7	1.9 ± 1.6	1.7 ± 1.4	1.2±1.3	2.2±1.7	0.8 ± 1.3	12.8 ± 7.1

**Table 4:** Significance of the OHIP-14 subscales according to city and regions.

	City *r*	Region *r*
Functional limitation	−0.028	−0.124**
Physical pain	−0.077	−0.052
Psychological discomfort	−0.010	−0.024
Physical disability	−0.019	−0.022
Psychological disability	0.070	−0.012
Social disability	0.058	−0.002
Handicap	−0.055	−0.033

**Significance of relationship at *P* ≤ 0.01.

**Table 5:** Comparison of DMFT scores and the MT component from similar population groups to the results of 3 OHIP-14 subscales in the present study.

	DMFT*	MT*	Functional limitation	Handicap	Psychological discomfort
Athens	**11.5**	**3.3**	**3.0**	**2.9**	**2.9**
Thes/niki	12.7	5.0	2.8	1.8	2.9
Patras	12.0	5.4	3.2	2.1	2.8
Ioannina	**16.9**	**7.1**	**2.4**	**1.9**	**2.8**
Kastoria	15.6	6.0	3.0	2.2	2.9

*Results presented were obtained from Oulis et al. 2009 [8].
